# Molecular iodine/doxorubicin neoadjuvant treatment impair invasive capacity and attenuate side effect in canine mammary cancer

**DOI:** 10.1186/s12917-018-1411-6

**Published:** 2018-03-12

**Authors:** Xóchitl Zambrano-Estrada, Brianda Landaverde-Quiroz, Andrés A. Dueñas-Bocanegra, Marco A. De Paz-Campos, Gerardo Hernández-Alberto, Benjamín Solorio-Perusquia, Manuel Trejo-Mandujano, Laura Pérez-Guerrero, Evangelina Delgado-González, Brenda Anguiano, Carmen Aceves

**Affiliations:** 1 Instituto de Neurobiología, Universidad Nacional Autónoma de México, Boulevard Juriquilla 3001, CP 76230 Querétaro, Mexico; 2 Facultad de Estudios Superiores Cuautitlán, Universidad Nacional Autónoma de México, Mexico City, Mexico; 3 Facultad de Ciencias Naturales, Universidad Autónoma de Querétaro, Mexico City, Mexico

**Keywords:** Canine mammary cancer, Molecular iodine, Neoadjuvant chemotherapy, Doxorubicin, Animal welfare

## Abstract

**Background:**

Mammary cancer has a high incidence in canines and is an excellent model of spontaneous carcinogenesis. Molecular iodine (I_2_) exerts antineoplastic effects on different cancer cells activating re-differentiation pathways. In co-administration with anthracyclines, I_2_ impairs chemoresistance installation and prevents the severity of side effects generated by these antineoplastic drugs. This study is a random and double-blind protocol that analyzes the impact of I_2_ (10 mg/day) in two administration schemes of Doxorubicin (DOX; 30 mg/m2) in 27 canine patients with cancer of the mammary gland. The standard scheme (sDOX) includes four cycles of DOX administered intravenously for 20 min every 21 days, while the modified scheme (mDOX) consists of more frequent chemotherapy (four cycles every 15 days) with slow infusion (60 min). In both schemes, I_2_ or placebo (colored water) was supplemented daily throughout the treatment.

**Results:**

mDOX attenuated the severity of adverse events (VCOG-CTCAE) in comparison with the sDOX group. The overall tumor response rate (RECIST criteria) for all dogs was 18% (interval of reduction 48–125%), and no significant difference was found between groups. I_2_ supplementation enhances the antineoplastic effect in mDOX, exhibiting a significant decrease in the tumor epithelial fraction, diminished expression of chemoresistance (MDR1 and Survivin) and invasion (uPA) markers and enhanced expression of the differentiation factor known as peroxisome proliferator-activated receptors type gamma (PPARγ). Significant tumor lymphocytic infiltration was also observed in both I_2_-supplemented groups. The ten-month survival analysis showed that the entire I_2_ supplementation (before and after surgery) induced 67–73% of disease-free survival, whereas supplementation in the last period (only after surgery) produced 50% in both schemes.

**Conclusions:**

The mDOX+I_2_ scheme improves the therapeutic outcome, diminishes the invasive capacity, attenuates the adverse events and increases disease-free survival. These data led us to propose mDOX+I_2_ as an effective treatment for canine mammary cancer.

## Background

Mammary cancer is the neoplasia with most incidences in intact female dogs [1]. Currently, surgery remains the mainstay of canine mammary cancer treatment, but several studies describe adjuvant chemotherapy as an approach that increases quality of life and disease-free survival [2–5]. In women, the use of systemic chemotherapy before local surgical excision, known as neoadjuvant therapy, may also reduce the extent of local surgery without jeopardizing patient survival and disease-free survival [6]. This practice is still scarce in dogs, but recent reports describe the use of this approach to transform unresectable tumors into resectable ones even though disease-free survival or survival time was not significantly longer when different chemotherapeutic drugs were used as monotherapy [7–9].

One of the most important considerations in chemotherapy treatment is the attenuation of toxicity and chemoresistant effects. Slow infusion is one of the most explored strategies to reduce toxicity. Doxorubicin (DOX), a widely used therapeutic drug, exhibited a direct correlation between its toxic effects and its peak plasma concentration. Rapid intravenous bolus results in higher peak levels, whereas slow infusion rates lead to a greater area under the curve associated with less toxicity. However, the pharmacokinetics of this drug could be different between species. In humans, slow infusion (1 to 6 h or more) exhibited less toxicity in cardiac tissue and other organs [10], whereas in rodents fast infusion (5 versus 30 min) seems to be better protection [11]. Related studies in canines were not available until now. A second strategy, more represented in canine studies, is the use of chemotherapeutic drugs in different schemes or pharmaceutical presentations (metronomic therapy, liposomes, nanoparticles) [12–15], specific molecules that act as inhibitors of growth factor receptors (toceranib phosphate) [16, 17], components that exacerbate immune anti-tumor responses (anti Her2, TNFα) [18, 19] or even natural products with adjuvant antineoplastic effects (Curcumin) [20].

In this regard, previous work by several research groups has shown that molecular iodine (I_2_) exerts antineoplastic effects on different cancer cells grown under basal, chemoresistant and stem (spheroid) conditions by activating re-differentiation pathways and reversing chemoresistance and epithelial-mesenchymal transition (EMT) [21–23]. Similarly, pre-clinic (immunosuppressed mice and pharmacologically induced rats) and clinic mammary cancer protocols have shown that I_2_ supplementation inhibits tumor growth, activates the anti-tumor immune system and exerts antioxidant actions that prevent the severity of side effects generated by antineoplastic drug toxicity [24, 25]. The antineoplastic actions of I_2_ include direct mechanisms exerting oxidant effects on the mitochondrial membrane and indirectly generating an iodinated lipid known as 6-iodolactone (6-IL), a specific ligand of peroxisome proliferator-activated receptor gamma (PPARγ) [26]. PPARs are ligand-activated transcription factors, and three subtypes have been identified (PPAR alpha, beta, and gamma). PPARγ is involved in lipid metabolism and has recently been shown to play a significant role in cell proliferation, differentiation, and apoptosis in many types of cancer [27]. The present study was designed to analyze the effect of I_2_ supplementation in two DOX administration schedules: standard (21-day cycles; 20 min infusion) versus modified (15-day cycles; 60 min infusion) regimens in canine patients with mammary cancer.

## Methods

### Patients

The study was performed to include at least between 4 and 10 dogs per group according to our previous studies in rodents [25]. We invited and registered 93 patients who gradually abandoned the protocol for various reasons. Some of them related to the side effects (although we were very attentive to their health and well-being, many owners did not accept the loss of hair or the temporary change of mood), or due to other difficulties (abandonment of treatment, death independent of cancer, pyometra, etc.). As the study was double-blind, the confirmation of the groups was revealed until the day of the surgery and it was not possible to re-adjust them. Twenty-seven intact female dogs with acceptable health and a diagnosis of mammary cancer finished the protocol. All procedures were performed at the Veterinary Hospital of the Universidad Autónoma de Querétaro and at the Veterinary Hospital of FES-Cuautitlán, UNAM. Additionally, all procedures followed the Animal Care and Use program (NIH, USA) and were approved by the Research Ethics Committee of INB-UNAM (Protocol #102).

### Clinical protocol

Cytological diagnostic of mammary cancer was performed by a sample of puncture fine needle aspiration. Eligibility criteria also included signed informed consent and normal hepatic, renal and cardiovascular function (general blood and urine analysis and electrocardiogram, respectively). A biopsy was performed in accepted dogs to confirm the cytological diagnosis and determine the tumor classification according to Goldschmidt et al. [28]. A dog health questionnaire was filled out by the veterinary surgeon and the dog owner before every chemotherapy cycle. Toxicity was graded using the Veterinary Cooperative Oncology Group - Common Terminology Criteria for Adverse Events (VCOG-CTCAE) [29]. The clinical signs considered in the present work were weight, vomitus, diarrhea, anorexia, lethargy, anemia and neutropenia. Distant metastasis disease was identified using ultrasound and thorax radiographs.

### Chemotherapy

The standard scheme (sDOX) comprises four cycles of DOX (30 mg/m^2^) administered intravenously for 20 min every 21 days, while the modified regimen (mDOX) consists in the same amount of chemotherapy infused for 60 min in four, 15-day cycles.

### Molecular iodine

Was prepared with Lugol solution (Golden Bell, Edo. de Mexico, Mx) and diluted in distilled water. Iodine (10 mg/day) or placebo (water with artificial food coloring) was administered orally with daily food 1 week before chemotherapy and continuously until mastectomy. The file for each dog was opened on the day of the mastectomy to identify the subgroup (I_2_ or placebo). To analyze the effect of I_2_ supplementation on survival and disease-free survival rates, all patients received the halogen supplementation for ten more months.

### Mastectomy

The veterinary surgeon carried out the pre-surgical protocol and the corresponding postoperative monitoring during the peri-mastectomy period. The surgery was performed 6 weeks after the last chemotherapy to ensure the complete re-establishment of the patients’ health. Anesthesia included premedication with 0.01 mg/kg Acepromazine (Vetoquinol, Mexico City, MX), induction and maintenance with 2 mg/kg Propofol (App pharmaceutics, Los Angeles, CA) and fluid therapy (saline solution). For pain management, 0.005–0.02 μg/kg with Buprenorphine (Schering-Plough, NJ, USA), 0.2 mg/kg Meloxicam (Aranda Lab, Queretaro, MX) and 1 mg/kg Tramadol (Pisa, Mexico City, MX), as well as antibiotic therapy with 5 mg/kg Enrofloxacin (Bayer, Edo de Mexico, MX).

### Iodine consumption, thyroid status, cardiac damage and tumor response

All these parameters were recorded on the day that the dogs were accepted into the protocol (initial) and on the day of the mastectomy (final). The content of total iodine in urine was determined by ionic analysis (potentiometer Thermo Scientific Orion Star A214 with ion selective electrode; ISE; LIS-146ICM), triiodothyronine (T3) and thyrotropin (TSH) circulating levels by ELISA (International immuno-diagnostics/IIDE-2021 and Abnova/KA2296, respectively). Serum creatine kinase type MB (CK-MB) activity, used as a marker of cardiac damage, was measured by using the CK-MB Test (Stanbio Laboratory/0980–103). Electrocardiogram (ECG) recordings were made over a minimum 30-s period in each dog, while conscious and lightly restrained, on initial (time 0), 10 min before each chemotherapy session and 1 week before mastectomy. The ECG traces from each animal were examined by a certified veterinary cardiologist to determine the following variables: heart rate, P-wave duration, P-wave amplitude, PR interval, QRS duration, QRS amplitude, QT interval, ST segment structure, and T-wave amplitude.

The tumor response rate was determined according to the Response Evaluation Criteria in Solid Tumors (RECIST) which use only the longest diameters of tumors in the axial plane [30]. The response is placed into one of four categories: complete response (CR; disappearance of all target lesions), partial response (PR; ≥ 50% decrease in sum of target lesions from baseline), stable disease (SD; meets criteria for PR or PD) or progressive disease (PD; ≥ 25% increase in sum of target lesions or appearance of new lesions).

### Immunohistochemistry

Was performed on 3 or 5 μm formalin-fixed paraffin-embedded specimens that were float-mounted onto silanized glass slides. ERα was analyzed by a sandwich protocol (polyclonal first antibody HC-20, Santa Cruz Biotechnology. Dallas, Texas, USA; and goat anti-rabbit-immunoglobulin, peroxidase labeled, Dako/K4011). Sections were counterstained with hematoxylin. ERα-positive cells were identified by the presence of a brown stain over the nucleus and cytoplasm. Five regions were analyzed, and tumor samples were considered ERα-positive when 20 or more cells per field were positively labeled in the nucleus, in the cytoplasm or in both, and were found in at least three fields at 40X. Negative controls were obtained by using only the secondary antibody (Dako/K4011). The positive control was performed employing ovary tissue.

### Histopathological analysis

Classification and grading of mammary tumors was determined by Goldschmidt et al. criteria which include tubule formation, nuclear pleomorphism and mitoses/hyperchromatism [28]. The histologic malignancy was described as well differentiated (low values), moderately differentiated (intermediate values) and poorly differentiated (high values). Hematoxylin and eosin (H&E) staining was used to observe histological malignancy and lymphocytic infiltration. Masson’s trichrome staining was used to identify epithelial (red) and connective (blue) proportions of representative slides per group. Two independent observers evaluated all these characteristics in anonymized and blinded samples. The analysis was performed as the mean of three random regions. The number of lymphocytes and the percentages of stained areas were calculated using the ImageJ 1.47 program (Wayne Rasband. NIH, USA).

### Molecular expression

Bax, Bcl2, urokinase plasminogen activator (uPA), Survivin, multidrug resistance protein 1 (MDR1) and PPARγ expression were analyzed by quantitative real-time PCR (qPCR) [21]. Total RNA was obtained using the TRIZOL reagent (Life Technologies, Inc., Carlsbad, CA), and the extracted RNA (2 μg) was reverse transcribed using oligo-deoxythymidine. qPCR was performed with SYBR green dye (Thermo Scientific/K0221) in detector system Roto-Gene 3000 (Corbett Research, Mortlake, NSW, Australia) with the canine gene-specific primers listed in Table 1. Validation of qPCR was performed using β-actin and phosphoglycerate kinase 1 (PGK1) as non-regulated housekeeping genes. Gene expression was calculated using the D cycle threshold method and normalized to β-actin content.

**Table 1:** Oligonucleotides

Gen	Reference	Forward primer	Reverse primer	pb
β-actin ^1^	NM_001101	acagagtacttgcgctcagga	ccatcatgaagtgtgacgttg	175
PGK1^2^	NM_053291.3	tgactttggacaagctggacgtga	cagcagccttgatcctttggttgt	110
Bax^3^	NM_001003011.1	aagctgagcgagtgtctcaagcgc	tcccgccacaaagatggtcacg	366
Bcl_2_^4^	NM_000633.2	gtggaggagctcttcaggga	aggcacccagggtgatgcaa	304
PPARγ^5^	XM_005632014.1	ttccattctcaagagcggaccc	tctccacagactcggcattcaa	190
Survivin^6^	NM_001003348.1	accgcgtctctacgttcaag	ccaagtctggctcgttctca	114
uPA^7^	XM_005618862.1	ttggggagatgaagtttgaggtgg	cagaacggatcttcagcaaggc	105
MDR1^8^	NM_001003215.1	tatcagcagcccacgtcatc	cagccactgctacctacgag	214

^1, 3, 4^ Human, ^2^ Rat, ^5, 6, 7, 8^ Canine

### Statistical analysis

All statistical analysis was performed in Prism version 6.01 (GraphPad Software). An unpaired, Student’s *t-*test was used to compare average (initial and final) of total urine iodine, serum thyroid hormones and cardiac enzyme (CK-MB). Unpaired one-way ANOVAs were used to analyze electrocardiogram profile, residual tumor size and epithelial/connective tissue proportion. The differential gene response was performed with Mann-Whitney U, and Pearson correlation was used to determine correlations between residual tumor size and lymphocyte number. A *p* value of less than 0.05 was considered significant.

## Results

Table 2 summarizes the patients’ characteristics. The median age was 9.2 ± 2.4 years, and the most affected breed was the Standard Poodle (33%). Sixty-eight percent of dogs were nulliparous, and none received hormone therapy. Only 20% (6 dogs) of the patients exhibited overweight (between 10% and 30% above standard weight). Two patients presented distant metastasis (lungs) at the time of diagnosis (clinical state V).

**Table 2:** Patient characteristics

Breed	Age (years)	Body weight (kg)	Clinical stage (TNM)ϕ	Number of tumors	Parturition	ERα
Standard scheme (Dox)						
Standard Poodle	12	6	III	2	0	Positive
Maltese bichon	9	**9.9**	III	7	2	Positive
Dachshund	13	6.6	III	4	2	Positive
Maltese bichon	7	**6.6**	III	5	0	Positive
Standard scheme (Dox + I_2_)						
Rottweiler	8	38	III	1	2	Positive
Standard Poodle	12	5.8	II	5	0	Positive
Standard Poodle	10	3.8	III	5	0	Positive
Labrador Retriever	10	30	III	7	0	Positive
Standard Poodle	12	6.4	V	8	1	Positive
Standard Poodle	9	7	III	9	1	Positive
Modified scheme (Dox)						
Cocker Spaniel	9	10.3	II	8	0	Positive
Mixed breed	6	4.2	II	2	0	Positive
Cocker Spaniel	8	13	I	6	3	Positive
Chihuahua	5	2.2	I	3	0	Positive
German Shepherd	7	30.1	III	7	0	Positive
Dalmatian	6	23	I	1	0	Positive
Modified scheme (Dox + I_2_)						
Cocker Spaniel	10	**16.8**	III	1	0	Positive
Mixed breed	8	**7.6**	I	5	0	Positive
Standard Poodle	11	4.2	V	7	0	Positive
Cocker Spaniel	13	9.8	III	7	0	Positive
Fox terrier Toy	7	2.4	I	5	0	Positive
Standard Poodle	10	4.5	III	3	0	Positive
Cocker Spaniel	13	**17.4**	III	7	3	Positive
Standard Poodle	10	6	II	8	0	Positive
Standard Poodle	9	4.7	III	6	4	Positive
Chihuahua	5	**3.8**	III	5	0	Positive
Dachshund	10	5.2	III	5	1	Positive

ϕ T – Primary tumor, N – Regional lymph nodes, M – Distant metastasis [28];

Bold numbers, overweight animals (10–30% of standard weight)

**Fig. 1:**
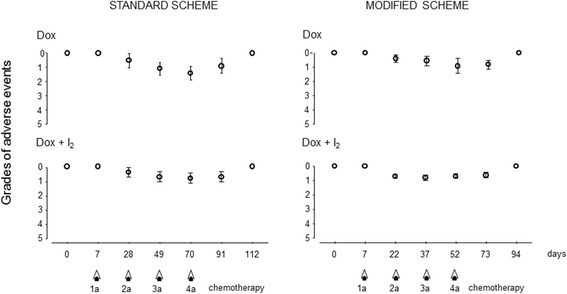
Functional value according to the VCOG-CTCAE scale. Each point represents the mean and SD for each clinical report during all treatments. Arrows represent the day of chemotherapy (DOX) application

Grading of adverse events for each group throughout the treatment is summarized in Fig. 1. Patients started with excellent conditions (0), and lower and medium grades (1 to 2) were observed after the second or third chemotherapy cycle (before the third or fourth chemotherapy cycle, respectively). No patients exhibited significant weight loss or developed grades 3, 4 or 5 (death) during treatments. Table 3 describes the number of patients who presented clinical signs and their severity (grade) at any time during chemotherapy treatment. The mDOX scheme reduces the severity of the general symptoms in comparison with sDOX. The I_2_ supplement further attenuates the severity of the symptoms in both treatments. The six dogs that presented grade 2 vomiting and/or diarrhea were complemented with supportive care: antibiotic (Metronidazole, 30–40 mg/kg for 14 days) and probiotics (Enterogermina 2 billion/5 mL); with Omeprazole (20 mg/kg for 24 h), Ranitidine (3 mg/kg every 12 h for 8 days), Difenidol (1 mg/kg for 4 days), and/or fluid therapy (saline solution).

**Table 3:** Adverse events (VCOG-CTCAE)

	Grade	sDox	sDox + I_2_	mDox	mDox + I_2_
Vomiting	1	1 (25%)	1 (17%)	1 (17%)	2 (18%)
	2	1 (25%)	1(17%)	1 (17%)	
	3				
	4				
Diarrhea	1	1 (25%)	1 (17%)	1 (17%)	2 (18%)
	2	1 (25%)	1 (17%)	1 (17%)	1 (9%)
	3				
	4				
Anorexia	1	2 (50%)	1 (17%)	1 (17%)	1 (9%)
	2	1 (25%)	1 (17%)		
	3				
	4				
Lethargy	1	1 (25%)	1 (17%)	1 (17%)	1 (9%)
	2	1 (25%)	1 (17%)	1 (17%)	1 (9%)
	3				
	4				
Anemia^a^	1	2 (50%)	2 (33%)	2 (33%)	3 (27%)
	2				
	3				
	4				
Neutropenia	1	1(25%)	1 (17%)	1 (17%)	1 (9%)
	2				
	3				
	4				

^a^Anemia includes: hematocrit, hemoglobin and erythrocyte values (mean globular volume and mean hemoglobin concentration)

**Fig. 2:**
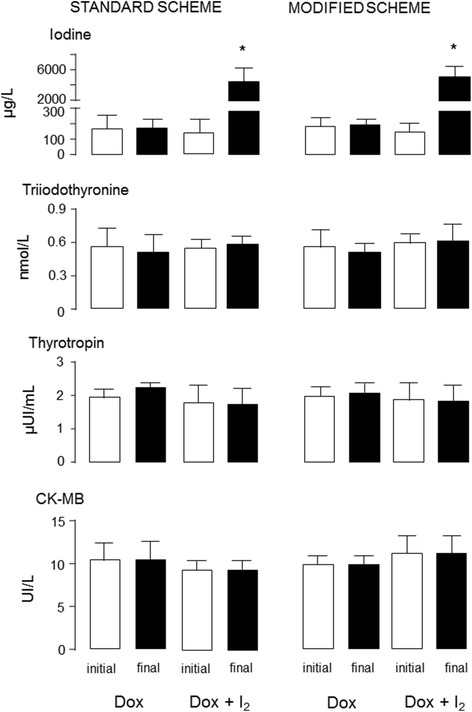
Iodine ingestion, thyroid status and cardiac damage during treatments. Values were recorded on the day of the patient’s admission to the protocol (initial) and on the day of mastectomy (final). Total iodine was determined in urine. Triiodothyronine, thyrotropin and creatine kinase type MB (CK-MB) were quantified in serum. Data are expressed as mean ± SD, and the asterisk indicates a significant difference with respect to the initial condition (unpaired Student t test; *P* < 0.05)

**Fig. 3:**
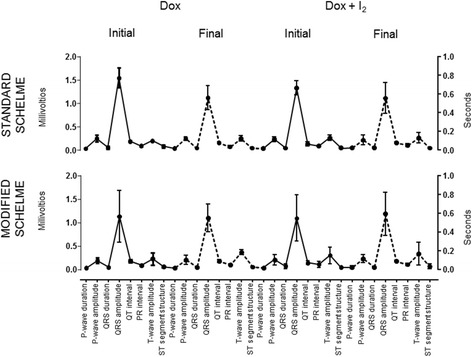
Electrocardiogram profile. Values represent the day of the patient’s admission to the protocol (initial) and 1 week before mastectomy (final). One-way ANOVA was performed for each variable and no significant differences were observed

I_2_ ingestion, thyroid status and cardiac damage were recorded when patients were accepted into the protocol (initial) and on the day of mastectomy (final). Total urinary iodine values were above 2000 μg/L in supplemented patients and DOX groups did not show any changes. I_2_ supplementation and/or DOX administration neither modified T3 or TSH circulating values or showed cardiac damage measured by serum CK-MB activity (Fig. 2). The electrocardiogram variables from initial (time 0) and final (before mastectomy) are summarized in Fig. 3. No significant changes were observed between groups in any variable or protocol day.

**Fig. 4:**
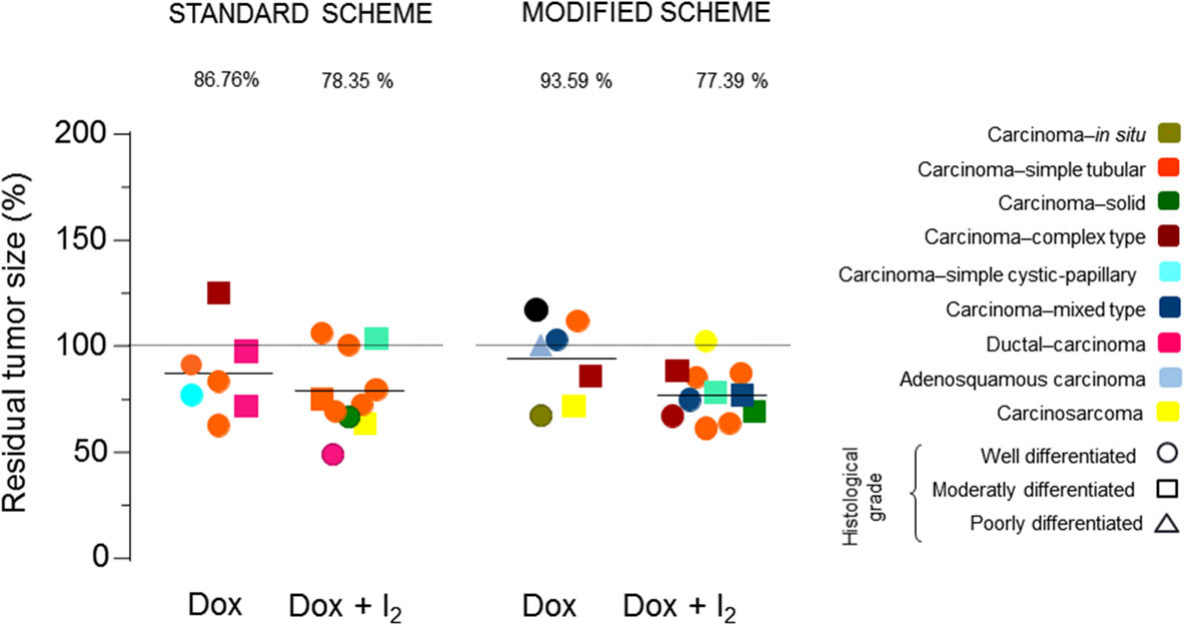
Residual tumor size (%), histopathological classification and grade of malignity were analyzed by RECIST scale. Each point represents an individual tumor. One-way ANOVA was performed, and no significant differences were observed

**Fig. 5:**
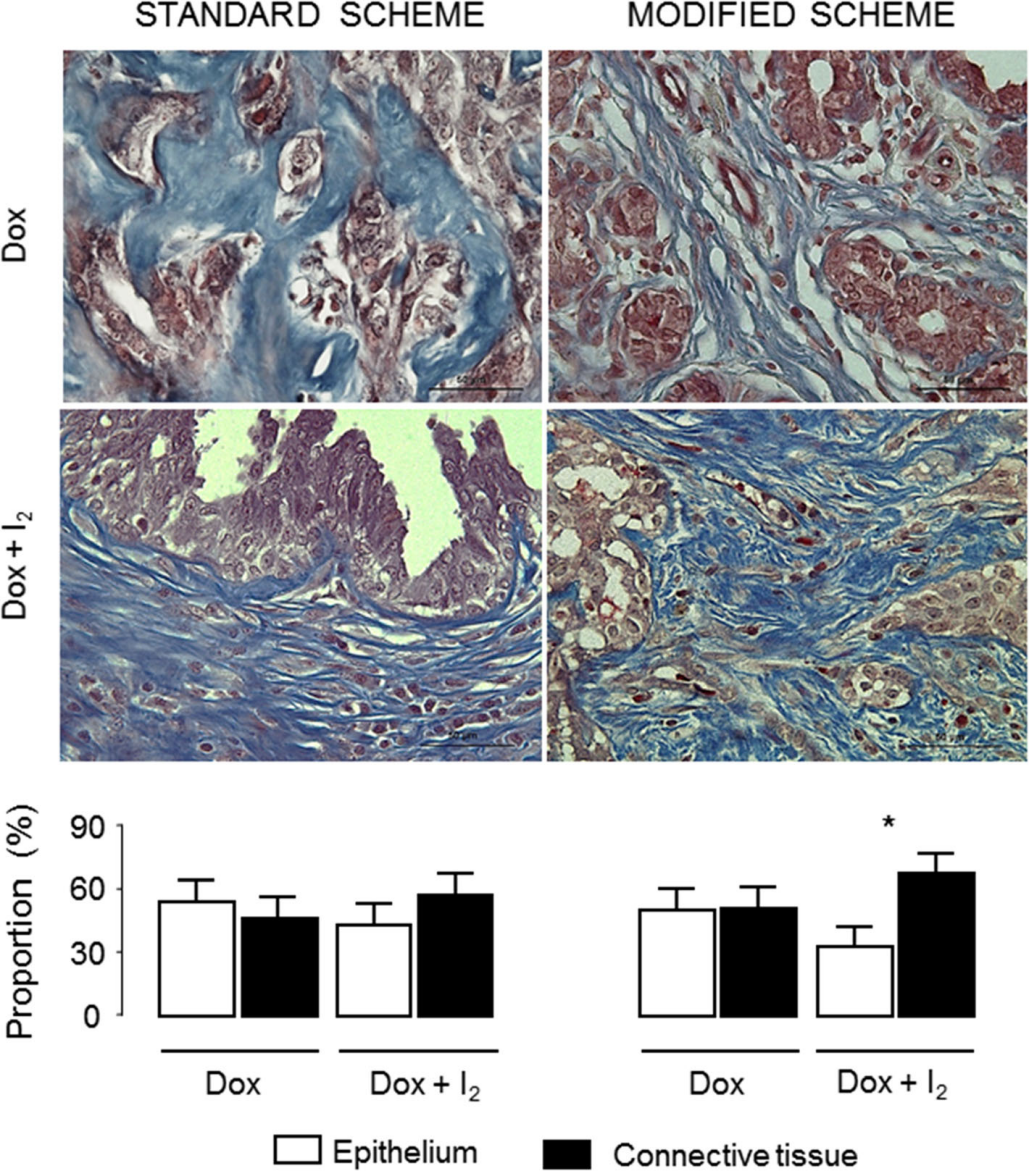
Epithelial and connective tissue proportion (%) in the final tumor mass. Micrograph staining with Masson’s trichrome method (red, epithelium; blue, connective tissue). Quantitative analysis was performed as the average of three random regions (40X) using the ImageJ 1.47 program. Data are expressed as mean ± SD, and the asterisk indicates a significant difference between groups. Student´s *t*-test (*P* < 0.05)

Figure 4 shows the tumor classification, histological grade and residual tumor size. Most patients developed more than one tumor (Table 2). The tumors with enough size (more than 1 cm^3^) were analyzed as individual tumors. Ninety-one percent were of the epithelial type, whereas only 9% corresponded to the mixed type (carcinosarcoma). Histological grading showed that 60% of tumors were well differentiated, 37% were moderately differentiated and only one (3%) was poorly differentiated. No dogs exhibited a complete response (CR). One patient from sDOX+I_2_ showed partial response (PR) and one from the sDOX group exhibited progressive disease (increases ≥25%). The rest of the tumors (94%) were maintained as stable disease. The overall response rate for all dogs was 18.0%, where the residual tumor size corresponds to 82.0% (interval 48–125%). However, even though tumor response (residual size) did not show statistical differences between groups, I_2_ supplementation was accompanied by decreases in epithelial tissue in comparison with connective content (Fig. 5). This observation was significant in the mDOX+I_2_ group, suggesting that I_2_ acts on all tumor types by increasing the antineoplastic effect of mDOX.

**Fig. 6:**
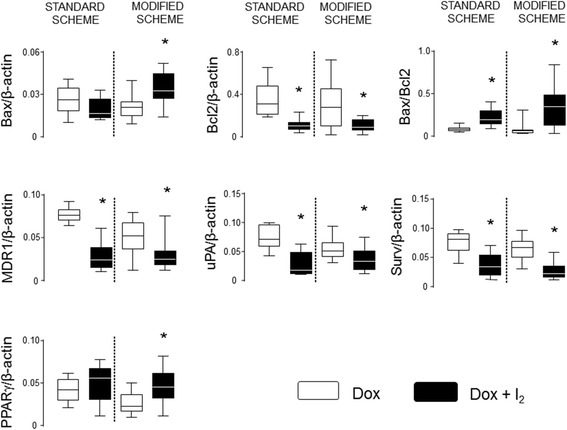
Effect of treatments on the expression of chemoresistant, invasive and differentiation markers. RT-qPCR amplification were performed in residual tumors. Bax/Bcl2 index as apoptotic induction. Multidrug resistance protein 1 (MDR1); urokinase plasminogen activator (uPA), Survivin protein (Surv). Peroxisome proliferator-activated receptors type gamma (PPARγ). Gene expression was calculated using the D cycle threshold method and normalized to β-actin content. Data are expressed as median and the asterisks indicate significant differences between DOX and DOX + I_2_ groups in each treatment (Mann-Whitney U; *P* < 0.05)

The molecular analyses (Fig. 6) show that I_2_ treatment generates similar responses in apoptotic and invasive markers independent of the DOX administration scheme. The presence of I_2_ increases the apoptotic index (Bax/Bcl2) and impairs the induction of invasive or chemoresistant genes such as MDR1, uPA and Survivin. The combination of mDOX+I_2_ synergies the expression of Bax and PPARγ receptors.

**Fig. 7:**
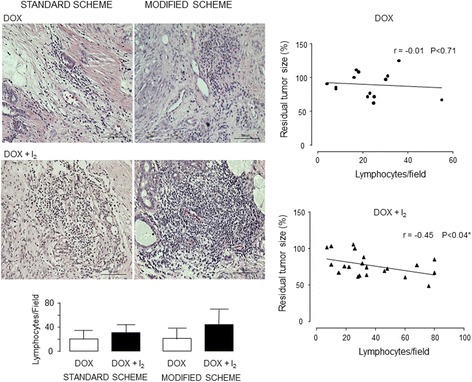
Effect of treatments on lymphocytic infiltration. Micrographs stained with H&E (20X). Quantitative analysis was performed as the average of three random regions using the ImageJ 1.47 program. Linear regression between residual tumor size (%) and lymphocyte number from DOX and DOX + I_2_ groups (* Pearson coefficient, *p* > 0.04)

One interesting phenomenon was observed in lymphocytic infiltration. I_2_ supplementation increases the presence of lymphocytes in both schemes (Fig. 7) and exhibits a significant inverse correlation with the tumor response (more lymphocytes in the small residual tumor size).

As mentioned before, the use of DOX as monotherapy has the benefit of converting the unresectable tumors into resectable ones, but it does not increase the disease-free survival (~207 days). With this premise, all our patients received I_2_ supplementation after the mastectomy, and the survival analysis was performed for ten more months (~300 days). Table 4 shows that I_2_ supplementation during the entire treatment (before and after surgery) has an improved disease-free survival of 67 and 73% (sDOX+I_2_: 4 dogs and mDOX+I_2_: 8 dogs, respectively) compared with the 50% (sDOX: 2 dogs; mDOX: 3 dogs) observed in those that received I_2_ only in the last period (after chemotherapy). The two patients with previous metastasis (lungs) received sDOX+I_2_ and mDOX+I_2_, each. In the first case, metastasis disappeared and after 10 months the patient was still alive without relapse or distant metastasis. In the second case (mDOX+I_2_), the metastasis progressed, and the patient died 4 months after surgery. Two patients (in the sDOX+I_2_ group) exhibited local mammary cancer relapse 7 months after surgery. Tumors were removed in a second surgery and after 10 months, both were still alive without evident metastasis. Three dogs died from causes independent of mammary cancer (two due to other cancers and one due to pyometra complications).

**Table 4:** Survival analysis

Breed	Age (years)	Clinical stage (TNM)	Survival at ten months	Observations
Standard scheme (Dox)				
Standard Poodle	12	III	Yes	Alive without relapse
Maltese bichon	9	III	No	Death, surgical complications
Dachshund	13	III	Yes	Alive without relapse
Maltese bichon	7	III	No	Death, surgical complications
Standard scheme (Dox + I_2_)				
Rottweiler	8	III	Yes	Alive with relapse; no metastasis
Standard Poodle	12	II	Yes	Alive without relapse
Standard Poodle	10	III	Yes	Alive with relapse; no metastasis
Labrador Retriever	10	III	Yes	Alive without relapse
Standard Poodle	12	V	Yes	Alive without relapse
Standard Poodle	9	III	Yes	Alive without relapse
Modified scheme (Dox)				
Cocker Spaniel	9	II	No	Death, transitional cell cancer
Mixed breed	6	II	No	Euthanasia, suspected metastasis
Cocker Spaniel	8	I	Yes	Alive without relapse
Chihuahua	5	I	Yes	Alive without relapse
German Shepherd	7	III	No	Death, anaplastic cancer
Dalmatian	6	I	Yes	Alive without relapse
Modified scheme (Dox + I_2_)				
Cocker Spaniel	10	III	Yes	Alive without relapse
Mixed breed	8	I	Yes	Alive without relapse
Standard Poodle	11	V	No	Death, extensive metastasis
Cocker Spaniel	13	III	Yes	Alive without relapse
Fox terrier Toy	7	I	Yes	Alive without relapse
Standard Poodle	10	III	Yes	Alive without relapse
Cocker Spaniel	13	III	No	Death, pyometra
Standard Poodle	10	II	Yes	Alive without relapse
Standard Poodle	9	III	Yes	Alive without relapse
Chihuahua	5	III	Yes	Alive without relapse
Dachshund	10	III	No	Death, surgical complications

## Discussion

In the present work, we compared two DOX administration schemes and the adjuvant effect of I_2_ in canine mammary cancer. Our results showed that both DOX schemes are well tolerated, since no patients exhibited grades 3 or 4, according to the VCOG-CTCAE [29], in both conditions. Moreover, cardiac damage was not evident through serum CK-MB activity or ECG in any group. The clear attenuation of adverse events observed in mDOX agrees with previous reports in humans where slow infusion (1 to 6 h) exhibited less toxicity [10], suggesting that the pharmacokinetics of DOX could be similar between humans and dogs. The addition of the I_2_ supplement appears to further attenuate the severity of adverse events. This benefit could be explained by the antioxidant effect of this chemical form of iodine, which is ten times more efficient than ascorbic acid and 100 times more potent than KI (Ferric reducing/antioxidant power assay; FRAP) in vitro [24]. Indeed, previous reports from our group in a rodent mammary cancer model showed that I_2_ supplementation prevented weight loss and cardiotoxicity (cardiac lipoperoxidation and serum CK-MB) secondary to DOX, without impairment of its synergistic antitumor action [24].

Regarding the tumor response (RECIST), the overall response was 18% showing that 94% of tumors were maintained as stable disease. This percentage of response is like that described by other authors when chemotherapy drugs are used in the form of monotherapy [6–8]; however, the important advantage observed in the I_2_-supplemented groups was the significant attenuation of invasive potential in the residual tumor mass. The tumor content showed a significant decrease in epithelial tissue, which is considered the source of tumor progression and metastasis, and a minimum expression of chemoresistance (MDR1 and Survivin) and invasion (uPA) markers, suggesting a better prognosis. It is well established that fibroblast and connective tissue are not I_2_-capturing tissues, whereas several types of epithelium uptake this halogen [31]. Moreover, it is described that cancerous cells exhibited between 10 and 100 times more sensitivity to apoptotic I_2_ effects than its normal counterpart [22, 31]. Two main pathways have been proposed to explain these effects; one concerning the direct oxidant/antioxidant action of I_2_ and a second pathway through iodolipid formation. Upadhyay et al. [32] showed that mitochondria isolated from the tumor (TT) and extra-tumoral tissue (ET) of human breast display significant uptake of iodine; but only TT mitochondria respond by increasing their permeability transition and releasing apoptogenic proteins, indicating a direct and differential proapoptotic action of I_2_ in mitochondria from cancerous cells. The second pathway is the generation of an iodinated derivative of arachidonic acid (AA), called 6-iodolactone (6-IL). AA is an essential membrane lipid, and its elevated concentrations in cancerous cells have been implicated in tumoral processes [33, 34]. 6-IL has been detected in tumor mammary glands from rats supplemented with I_2_ [35], and this iodolipid is a specific agonist of PPARγ [36]. Treatment with PPARγ agonists inhibits cancer cell growth by inducing G_0_–G_1_ cell-cycle detention, promoting differentiation and reverting the EMT [37]. EMT is characterized by the suppression of adhesion molecule expression (e.g., E-cadherin), the induction of mesenchymal proteins such as N-cadherin or Vimentin and the acquisition of chemoresistance by up-regulation of ATP-binding cassette (ABC) transporters (such as MDR1) and anti-apoptotic markers like Bcl2, Bcl-xl or Survivin [38]. Our observation that I_2_ supplementation in both schemes diminishes part of these markers (MDR1, Bcl2, uPA and Survivin) and increases the expression of PPARγ agrees with the notion that I_2_ exerts its antineoplastic effects through PPARγ activation. Moreover, the loss of invasive capacity of remnant cancerous tissue could explain the low rate of recurrence and metastasis found in our patients even after 10 months.

Another important finding was the significant inverse correlation of lymphocytic infiltration with the tumor response (residual tumor size) in samples from dogs treated with I_2_ supplementation. Previous reports described that the presence of lymphocytic infiltration in breast cancer tissue predicts a positive response of neoadjuvant chemotherapy, and led to speculate that the inclusion of conventional chemotherapy with sensitization of the immune system could be a promising paradigm [39, 40]. The specific mechanism of I_2_ in this putative immune modulation has not been elucidated, but it is demonstrated that several immune cell types can internalize I_2_, which can act as an anti- or pro-inflammatory agent depending on the cellular context [41, 42]. I_2_ can have anti-inflammatory effects either by suppressing the production of toxic oxygen intermediates in polymorphonuclear cells or by inhibiting neutrophil chemotaxis [43, 44]. In contrast, I_2_ may act directly on immune cells by inducing their reactivation. In chronic wounds, its presence activates the influx of macrophages and T cells [45]. In vitro, I_2_ enhances TNFα secretion from macrophages stimulated with bacterial lipopolysaccharides [46]. We observed a similar anti-tumor immune response in mammary cancer xenografts when mice Fox1 nu/nu were supplied with I_2_ (data not published). Moreover, PPARγ activation could be associated with such effects. In a recent publication, it was demonstrated that PPARγ expression increased almost 5-fold in a metronomic cyclophosphamide regime, suggesting that these receptors substantially contribute to the immune responsiveness of this treatment [47].

Finally, we wanted to test the effect of I_2_ supplementation on disease-free-survival and survival when DOX is applied as monotherapy. There are only three previous reports that analyze this parameter using neoadjuvant chemotherapy in canines [6–8]. They found that survival time could be significantly extended with combination therapies (two or three drugs plus radiotherapy), rising the disease-free survival mean to 12 months (interval 213–521 days). In contrast, the disease-free survival only lasts 7 months (217 days) when DOX is used as monotherapy [7]. Our study included a ten-month (~300 days) analysis, which showed that during this time overall disease-free survival was of 62%. I_2_ increases the patient’s survival when it is supplied from the beginning of treatment (67% in sDOX+I_2_ and 73% in mDOX+I_2_), and by 50% in both treatments when it is provided only after surgery. In the first case, I_2_ could act on two levels; first, by increasing the antineoplastic effects of DOX at the tumor site (differentiation induction, though less invasive phenotype and/or activation of immune response) and second, as an antioxidant, diminishing the oxidative damage in normal tissues (digestive, anemia, etc.) caused by DOX. The benefit after surgery (impaired relapse) could be linked to the antiproliferative effect of I_2_ per se. Several studies have reported that this antiproliferative action of I_2_ in mammary or prostatic pathologies (hyperplasia, mammary gland fibrosis or cancer) is useful only if the I_2_ supplement is maintained at moderated high concentrations, whereas its suppression resumes pathology progression [48]. These effects agree with epidemiological studies showing that Japanese population that decreased I_2_ consumption increased the incidences in mammary and prostatic pathologies [49].

## Conclusions

The combination of DOX + I_2_ in the modified scheme improved the therapeutic outcome of mammary cancer, increasing both survival rate and relapse-free survival.

## Abbreviations

6-IL: 6-iodolactone
ANOVA: Analysis of variance
DOX: Doxorrubicin
ECG: Electrocardiogram
EMT: Epithelial-mesenchymal transition
H&E: Hematoxylin and eosin
I_2_: Molecular iodine
mDOX: Modified scheme
MDR1: Multidrug resistance protein 1
PPARγ: Peroxisome proliferator-activated receptors type gamma
qPCR: Quantitative real-time PCR
RECIST: Response evaluation criteria in solid tumors
sDOX: Standard scheme
Surv: Survivin
T3: Triiodothyronine
TSH: Thyrotropin
uPA: Urokinase plasminogen activator
VCOG-CTCAE: Veterinary co-operative oncology group – common terminology criteria for adverse events
